# Scintillation in Liquid Xenon for Gamma-Ray Medical Imaging: From Single Time-over-Threshold to Multi-Time-over-Threshold PMT Signal Measurements

**DOI:** 10.3390/s24175826

**Published:** 2024-09-08

**Authors:** Quentin Lainé, Nicolas Beaupere, Dingbang Cai, Eric Morteau, Fabrice Seguin, Dominique Thers, Cyril Lahuec

**Affiliations:** 1 Lab-STICC, Optics Department, IMT-Atlantique, CS 83818, Cedex 3, F-29238 Brest, France; quentin.laine@imt-atlantique.fr (Q.L.); fabrice.seguin@imt-atlantique.fr (F.S.); 2 Subatech, IMT-Atlantique, CNRS/IN2P3, Université de Nantes, F-44000 Nantes, France; beaupere@subatech.in2p3.fr (N.B.); cai@subatech.in2p3.fr (D.C.); eric.morteau@subatech.in2p3.fr (E.M.); dominique.thers@subatech.in2p3.fr (D.T.)

**Keywords:** multi-time-over-threshold, Compton camera, scintillation light, three-gamma imaging, medical imaging

## Abstract

In this paper, a new light event acquisition chain in a three-gamma liquid xenon prototype for medical nuclear imaging is presented. The prototype implements the Multi-Time-Over-Threshold (MTOT) method. This method surpasses the Single-Time-Over-Threshold (STOT) by precisely determining both the number of vacuum ultraviolet (VUV) photons detected by each photomultiplier tube (PMT) and their arrival times for light signal measurement. Based on both the experimental and simulated results, the MTOT method achieved a 70% improvement in reconstructing photoelectrons (PEs) and enhanced the precision of the arrival time estimation by 20–30% compared with STOT. These results will enable an upgrade of the XEMIS2 (Xenon Medical Imaging System) camera, improving its performance as the imaged activity increases.

## 1. Introduction

XEMIS2 is a monolithic Compton camera using liquid xenon (LXe), designed for imaging small animals with a low activity via a specific radionuclide employing the three-photon imaging technique [1]. The camera is based on an LXe Time Projection Chamber (TPC), detecting both the scintillation and ionisation signals that result from the interaction of the γ rays with LXe. It enables the direct localization in three dimensions of the radionuclide. The initial objective of the XEMIS testing phase is to replicate the image quality of Time of Flight Positron Emission Tomography (TOF PET) systems while using 100 times less injected activity. TOF PET systems typically achieve millimeter-level resolution with activities on the order of MBq [2]. XEMIS aims to achieve this same quality in a preclinical whole-body examination of a small animal in 20 min with an injected activity of only 20 kBq [3]. To maintain the same image quality while reducing the clinical examination time to 2 min, it is necessary to increase the administered activity to 200 kBq and reduce the LXeTPCs occupancy rate. An upgrade of the XEMIS2 camera could reach this target.

Dedicated PMTs, in the XEMIS2 camera, collect the VUV LXe scintillation photons and convert them into a number of PEs proportional to the detected photons [4]. The primary purpose of the scintillation signal is to measure the interaction time of the gamma rays in liquid xenon. Virtual segmentation of the fiducial volume is used to reduce the occupation rate of LXeTPCs. By measuring the average number of PEs produced by the scintillation signal and collected by the PMTs, preliminary spatial pre-localisation of the gamma interactions within the virtual LXe segmented volumes allows for more effective association of the scintillation and ionisation signals [5].

In the XEMIS2 camera, the total number of PEs per PMT measured in the LXeTPC can be quite large (in the order of a few hundred), and the light signal acquisition electronics are not precise enough to estimate it with accuracy. With the goal of pre-locating interactions, an upgrade of the XEMIS2 electronics for reading and acquiring the light signal is necessary to achieve complete coverage of the detection zone by PMTs. This involves increasing the number of PMTs and improving the light signal data acquisition system (DAQ). In this article, work on improvement of the light signal DAQ system is presented. In this context of an upgrade, a new measurement system based on the MTOT method is developed to better assess the number of PEs measured by each PMT. The scintillation signal in XEMIS2 and its current DAQ system are introduced in Section 2. Section 3 presents an initial prototype electronic board using MTOT with four different threshold levels and its calibration on a test bench. Based on the results obtained with the prototype, the final section includes an MC simulation to extrapolate the prototype results to real experiment conditions.

## 2. XEMIS2 Camera

**Figure 1:**
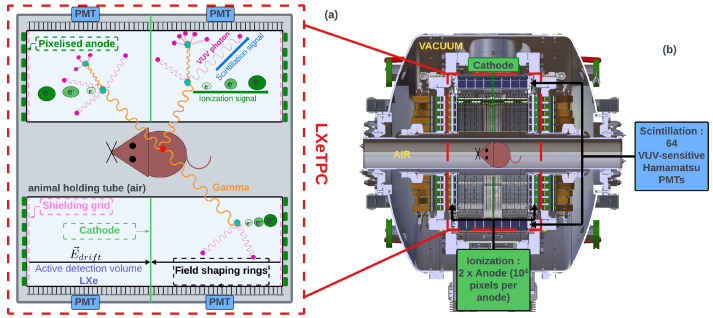
Schematic diagram of the principle of XEMIS2. (**a**) Operating principle of the LXETPC for a three-gamma event. Gammas interact with LXe through Compton scattering or the photoelectric effect. (**b**) Internal schematic view of the XEMIS2 detector in a longitudinal view.

The camera consists of two identical cylindrical LXeTPC (Figure 1). They are positioned back to back and share the same cathode located at the center of the detector. With this geometry, the detection volume of each LXeTPC is optimized for the simultaneous detection of the three γ-rays with a high positional sensitivity. When a γ-ray emitted from the small animal interacts in the detector cryostat, LXe generates scintillation photons in the VUV region and ionization charge carriers simultaneously. Scintillation photons are emitted isotropically within a 4π solid angle, propagate through the LXe, and are then collected by 64 VUV-sensitive PMTs that cover the detection volume. The fast emission and high propagation speed of scintillation light in LXe suggest that the arrival time of scintillation photons at the entrance window of PMTs is equivalent to the event’s initial time. The charge carriers are drifted by a uniform electric field and collected by two segmented anodes placed on both sides of the LXeTPCs. A shielding Frisch grid is used to mitigate the dependence of the ionization signal on the drift time and the longitudinal position of the interaction, ensuring more accurate and uniform signal detection. Each anode is segmented into 10,000 pixels, each measuring 3 × 3 mm^2^, to measure the transverse coordinates, the deposited energy and the drift time, for each interaction vertex. The longitudinal coordinate (along the drift direction) can be inferred from the electron drift time, which is the time delay between the scintillation signal and the ionization signal, and the electron drift velocity inside LXe. Identification of gamma-rays is feasible by utilizing both the deposited energy and the spatial position per interaction vertex.

### Scintillation Signal Read-Out and Data Acquisition System

PMTs collect the VUV photons and convert them into PEs, which are then amplified by the PMT system to generate a measurable analogue pulse proportional to the number of PEs. Each PMT is connected to the acquisition electronics, which are located outside the detector. The light DAQ system comprising four functional units, the Hamamatsu R7600-06MOD-ASSY PMT, dedicated to detecting VUV photons in LXe [6], a front-end signal reading electronic board, referred to as XSRETOT (XEMIS Scintillation Read-out for Extraction of Time Over Threshold), and an FPGA acquisition board (Xilinx SPARTAN-6 LX 150, Xilinx, San Jose, CA, USA) used for digital conversion, serialization, and transmission of data to be stored on the acquisition computer.

The XSRETOT board consists of a 16-channel low-pass RLC Pulse-Shaping Amplifier (PSA) to shape and amplify the analogue pulses delivered by the PMT, and one Leading Edge Timing Discrimination (LETD) module per channel [5]. Each channel of the PMT is auto-triggered by the LETD module at a threshold voltage calibrated to record at least 1000 noise signals per second. While the analog pulse from the PSA exceeds this threshold voltage, a gate-shaped signal, called the time over threshold (TOT) signal, is produced by the discriminator. The duration of the TOT signal represents the time over the threshold of the PSA signal, and its start time, known as the leading edge (LE), is determined by the threshold crossing by the rising edge. LE and TOT for each channel are then read and processed by the FPGA. The number of PEs and the interaction time of the γ rays are extracted from TOT and LE, respectively [4]. It is important to note that the deposited energy in LXe is extracted not from the scintillation signal, but from the ionisation signal. The TOT method improves the evaluation of the interaction time through an estimation of the number of PEs [5].

## 3. XSREMTOT: The Multi-TOT Protoype

In XEMIS2, the results of the Light Collection Map (LCM), representing the number of PEs measured ($NPE_{m}$) for each PMT, showed that in the central region of the LXeTPC, near the PMT array, the number of PEs can reach up to 150 [7]. The STOT method exhibits limitations under these conditions, particularly displaying non-linear behavior when measuring a high number of PEs. To achieve accurate spatial pre-localization of events, it is necessary to improve the precision of $NPE_{m}$. To address the intrinsic limitations of TOT, advanced techniques based on the same principle have been developed, such as Dynamic Time-Over-Threshold (DTOT) [8], which uses a variable dynamic threshold, and MTOT [9], which employs multiple fixed thresholds at different values to precisely reconstruct the charge from a known signal shape.

MTOT, in particular, has shown a good performance when reconstructing the charge of signals detected by PMTs, with, for example, a 20% resolution demonstrated in the KM3NET experiment [10]. However, the shape of the discriminated signal directly influences the precision of $NPE_{m}$. Due to electronic fluctuations (noise and PMT gain variation) and physical factors (delay induced by scintillation mechanisms in LXe), the PSA signal shape fluctuates significantly, leading to highly dispersed TOT values for the same charge. This necessitates the use of a specific reconstruction method for $NPE_{m}$.

**Figure 2:**
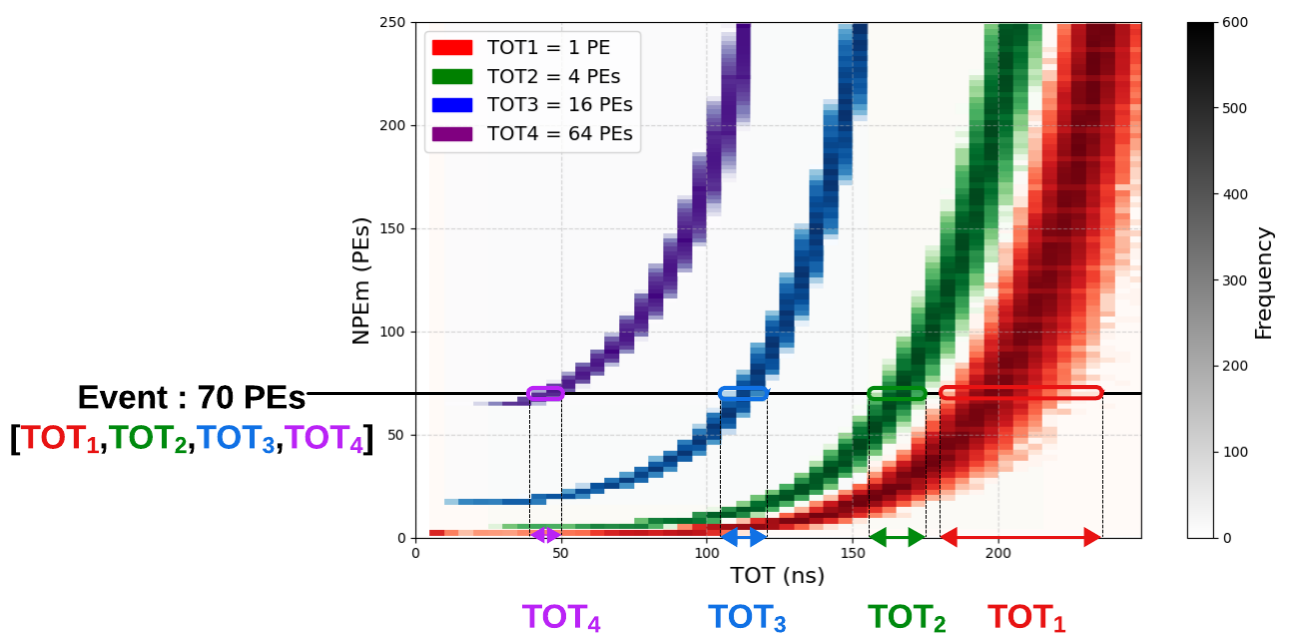
Experimental distribution of the $NPE_{m}$ according to the TOT for 4 different thresholds (N from 1 to 4). The four observed level of the thresholds are: 1, 4, 16, and 64 PEs. This figure illustrates how each threshold influences the distribution of the measured NPEs as a function of $TOT_{i}$.

Figure 2 illustrates this variation in TOT values based on test bench measurements, where only the variation induced by the electronics is represented. This figure shows the experimental distribution of $NPE_{m}$ measured as a function of TOT for four different thresholds (1, 4, 16, and 64 PEs). Each curve illustrates the relationship between TOT and $NPE_{m}$ for a given threshold, highlighting the non-linear evolution of $NPE_{m}$ with a saturation behavior at high TOT values, which depends on the threshold height. In the example shown, TOT values corresponding to a charge of 70 PEs can vary considerably. For a low threshold of 1 PE (red curve), after selecting the necessary physical events and the corresponding threshold used in the STOT method, TOT values can vary between 180 and 230 ns. However, this variation decreases for higher thresholds, with TOT values ranging from 155 to 175 ns for a 4 PE threshold (green curve), from 105 to 120 ns for a 16 PE threshold (blue curve), and from 45 to 50 ns for a 64 PE threshold (purple curve). The reduced dispersion for higher thresholds enhances the precision of the information provided by these thresholds. By combining the 4 TOT values of a single event ([$TOT_{1}$, $TOT_{2}$, $TOT_{3}$, $TOT_{4}$]), the most probable value of $NPE_{m}$ for this combination can be calculated with a high accuracy. Repeating this method for all combinations establishes a conversion table, as presented in the final section of this article.

However, it is important to note that increasing the number of thresholds requires adapting the DAQ system to handle the increased data volume. As XEMIS2 is designed to operate at low activity levels, the volume of light data remains relatively low, at around 100 MB/s per decay. The DAQ system can thus be easily adapted by serializing the four thresholds and increasing the number of acquisition boards.

**Figure 3:**
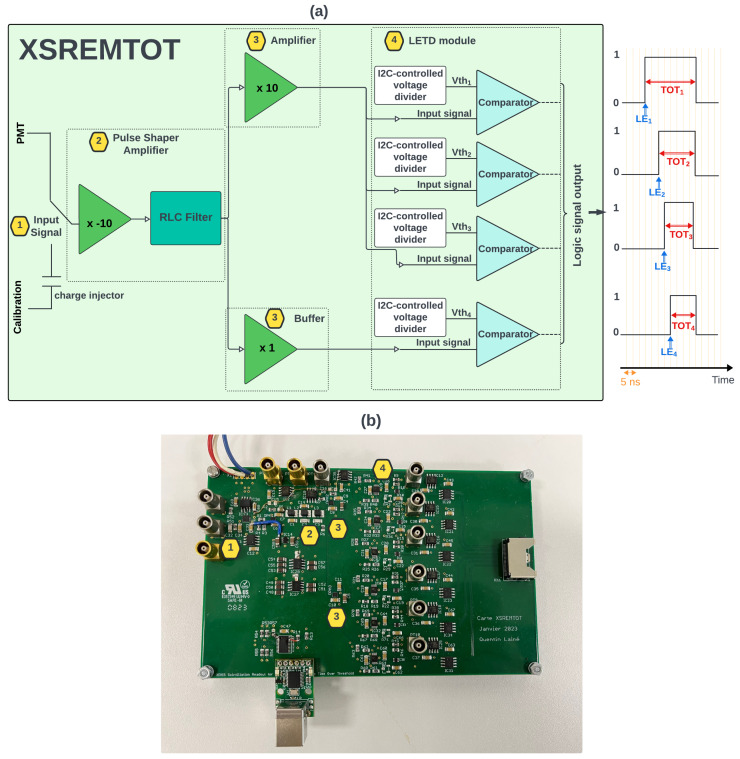
XSREMTOT board (**a**) and actual prototype (**b**). Numbers in the yellow hexagon provide localization of the block diagram onto the board.

A new prototype, which will replace the actual XSRETOT board and implement the MTOT method, has been carried out for integration into the upgraded XEMIS2 DAQ system. The new prototype front-end electronic signal reading board, named XSREMTOT (for XEMIS Scintillation Readout for Extraction of Multi-Time-Over-Threshold), aims to precisely measure the number of PEs using the MTOT method, as shown in Figure 3. This enhancement is intended to improve the accuracy of the arrival time measurement of the scintillation signal and to enable the spatial pre-localisation of physical events in the future. The board is designed with four separate threshold stages implementing the MTOT method. The operation of the integrated XSREMTOT card within the DAQ system is depicted in Figure 3a, and the actual prototype is shown in Figure 3b.

### 3.1. Board Description

This circuit, located outside the detector, after the PMTs in the acquisition chain, includes a prototype board with two input channels: PMT and calibration (first yellow hexagone in Figure 3). A wideband low-noise operational amplifier (OPA856; Texas Instruments, Dallas, TX, USA) is used to invert and increase the signal-to-noise ratio of the input signal. A PSA is then used to shape the PMT output pulse into Gaussian-like form (second yellow hexagone in Figure 3). This consists of a 6th-order low-pass RLC filter, inspired by work carried out on the ATLAS Tile Calorimeter [11]. At the output of the shaper filter, a second amplifier is used to increase the signal-to-noise ratio of the PSA signal for the first three threshold stages. For the last threshold, it is not necessary to amplify the PSA signal, as it is designed for large values of PE. However, a follower amplifier is used to isolate the channel and prevent the current at the comparator input from flowing back up to the other stages (third yellow hexagone in Figure 3). Four high-speed comparators (TLV3603; Texas Instruments, Dallas, TX, USA) are used to produce a gate shaped signal containing the TOT and LE (four yellow hexagone in Figure 3). Each comparator is connected to an independent I2C-controlled digital potentiometers to set the threshold voltage applied to the PSA signal. The board thresholds allow for self-triggering the PMT signals to a dedicated band-width. The main characteristics of the XSREMTOT prototype board are summarized in Table 1. The output signals are routed to the FPGA board, through LVDS lines.

### 3.2. Board Calibration

**Figure 4:**
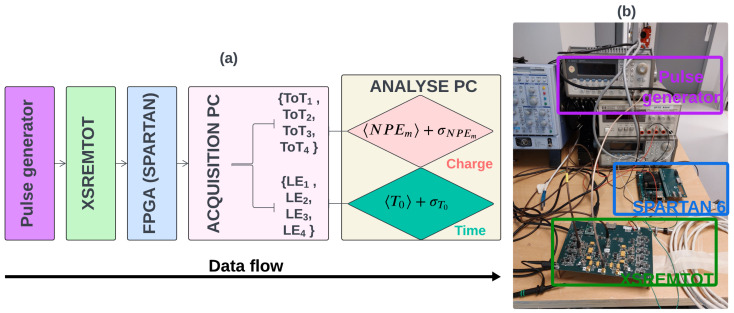
Test bench for the XSREMTOT board calibration protocol: (**a**) block diagram, (**b**) photo of the actual test bench installed in Nantes University Hospital.

A calibration protocol was carried out on a test bench to define the value of the four thresholds to reach a noise count rate of 1 kHz. The calibration set-up consists of a pulse generator, the XSREMTOT board, an FPGA, and a computer for data recording, as represented in Figure 4.

**Figure 5:**
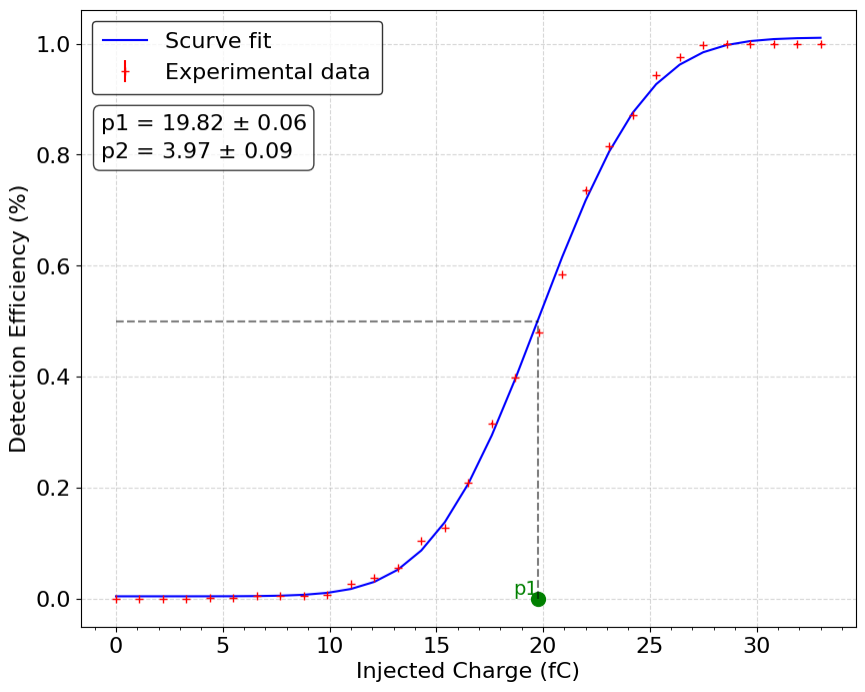
S-curve for a threshold of 1 PE. The parameter p1 corresponds to the charge injected for the desired threshold value when the S-curve fit reaches 50% of its maximum. Parameter p2 corresponds to the standard deviation of the fit.

The generator and capacitor injection on the calibration channel modeled a PMT signal. The S-curve fit method [12] is used to convert the threshold arbitrary unit to a voltage value and finally to a number of electrons. This number provides the PMT gain value to be set to detect 50 % of 1 PE signal. The first threshold is set at 1 PE, which acts as the initial veto between background noise and the light signals. The charge injected for this threshold value can be determined using the pulse and the injection capacitor (2.2 pF). This charge corresponds to the number of PMT gain electrons ($N_{e^{-}}=C\times \Delta V\times e^{-1}$, with e the elemental charge of an electron) required to produce a PSA signal equivalent to 1 PE. Figure 5 represents the first threshold calibration curve of the XSREMTOT board by setting its level to the one of 1 PE PSA signal. The curve shows the detection efficiency (the ratio between the number of detected events and the total number of injected events) according to the injected charge. As the electronic noise of the PSA signals follows a Gaussian distribution, the dependency of the detection efficiency on the threshold takes the form of a Gaussian error function, with the mean corresponding to the threshold value. The injected charge corresponding to this threshold is 19.82 fC (fit parameter p1). This represents around 125,000 electrons. The other three thresholds are then set at multiple values of this charge: 4 PEs, 16 PEs, and 64 PEs. The second threshold at 4 PEs is set to guarantee accuracy on small signals (<5 PEs), which will be the majority in the context of real-condition acquisition by the XEMIS2 camera. The third and fourth thresholds are defined to maximise accuracy on the highest amplitude signals throughout the interval studied (from 1 to 200 PEs). The S-curves show that the pulse shaping amplifier has a low noise of 3.97 fC as well (p2 fit parameter), which is equivalente to 1.8 mV.

### 3.3. Experimental Calibration Limitation

Experimental calibration on a test bench only provides an approximation of how the card operates under real conditions and does not faithfully represent the physical signals measured in XEMIS2. The calibration signal is generated by a waveform generator, which emits the input signals periodically, synchronized with its own frequency. In contrast, the actual emission of photons in LXe follows an exponential density law, meaning that photons are emitted asynchronously.

## 4. Asynchronous Study

**Figure 6:**
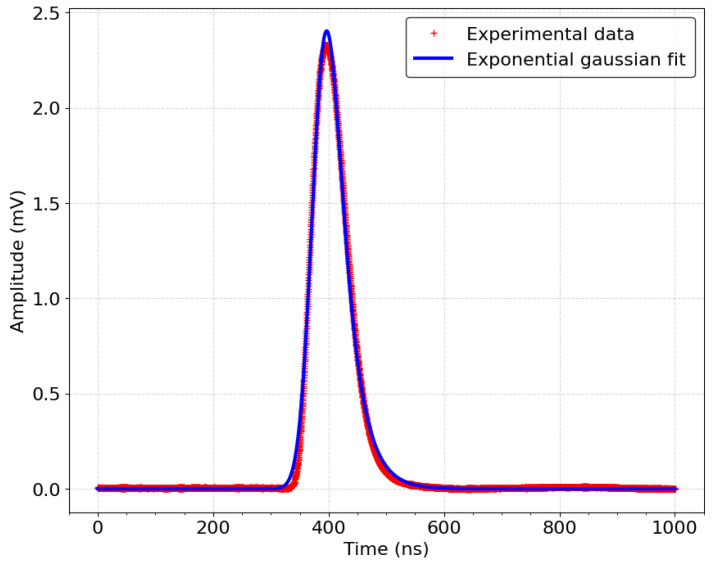
Experimental PSA signal for a charge equivalent to 1 PE (red cross) and Gaussian exponential model used for simulations (blue curve).

The aim of this study is to simulate the impact of the asynchronous scintillation photons emission on the PSA signals in order to accurately determine the number of measured PEs and the emission time of the light signal. The acquisition chain, from the PMT to the output of the PSA module, is modeled by fitting the experimental PSA signal at 1 PE, as shown in Figure 6. For this equivalent injected charge, the PSA signal output from the shaper filter is recorded and averaged over 100,000 signals, aiming to produce a realistic PSA signal representing 1 PE. The results show a PSA signal corresponding to a 1 PE charge with a peak amplitude of approximately 2.3 mV and a duration of around 150 ns, including a 50 ns rise time, matching the expected parameters for the filter design. A fit on the experimental data was performed using an exponential gaussian function. The remainder of the simulation is based on a high-performance model, incorporating four different threshold levels as well as the noise affecting the signal.

### 4.1. Noise Simulation

The asynchronous delay between each scintillation photons is reproduced by generating a random time for each PE. Each are randomly drawn according to one of three exponential distributions representative of the different types of VUV photon emission in LXe. The three types of scintillation photon emission in liquid xenon are fast, slow, and recombination, defined by their respective probabilities (3% for the fast component, 60% for the slow component, and 37% for the recombination component, for an electric field of 2 kV/cm applied in the LXeTPC [5]) with decay rates of 2.2 ns [13], 27 ns [13], and 45 ns [14], respectively. The PMT gain value varies by 50 % for each PE [7]. This variation is mainly due to the PMT dynode system used in the detector. In the simulation, this variation is applied to each PE signal by drawing a coefficient according to a normal law N(1,0.5). The signal variation induced by electronic noise is modeled by a random additive constant generated from a normal distribution with a standard variation equivalent to parameter p2 in Figure 5. This variation is then added to the completed PSA signal. Fluctuations in the sampling frequency are added to the calculated TOT values to account for temporal inaccuracies. Uncertainty in the TOT recording is modeled as a Gaussian distribution with a standard deviation of $5/\sqrt{12}$ ns. This value reflects the resolution of the timing mechanism and the impact of random errors on the TOT measurement, making the simulation of more realistic.

### 4.2. XSREMTOT Simulation

In the simulation, events ranging from 1 to 200 PEs are studied. The PSA signals are generated as a successive sum of delayed 1 PE signals and noise. To reproduce the operation of the XSREMTOT board, initial thresholds are defined at values of 1, 4, 16, and 64 PEs. TOT values ($TOT_{1}$, $TOT_{2}$, $TOT_{3}$, and $TOT_{4}$, respectively) are computed when the PSA signal exceeds each of these thresholds. The LE of each TOT is also computed ($LE_{1}$, $LE_{2}$, $LE_{3}$, and $LE_{4}$ respectively). Two sets of thresholds were defined for the simulation. The first set, 1-4-16-64, was modeled to replicate the same set used in the experimental setup. A second set, 1-4-8-32, was tested with reduced intervals between the thresholds to maximize precision over a smaller range.

### 4.3. Charge Conversion

**Figure 7:**
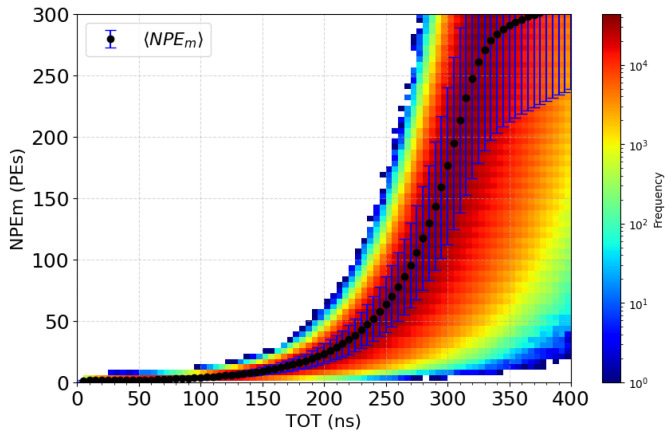
Distribution of the $NPE_{m}$ for different $TOT_{i}^{1}$ values, with the first threshold set to 1 PE.

According to the DAQ simulation, the link between the NPE, as the input of the simulation, and the set τ of possible TOT values, $\tau =\{\tau_{i}=\left\{TOT_{i}^{j}\;|\;j\in N,j\in \left[1,4\right]\right\}\;|\;i\in N\}$, as the output of the simulation, is known. A conversion table can be built to obtain the reverse link: measuring a set of TOT ($\tau_{i}$) determines the number of measured PEs ($NPE_{m}$). However, according to the noise, a same set $\tau_{i}$ can provide different values of $NPE_{m}$. Therefore, for each NPE, 100,000 repetitions of the simulation are processed to obtain the distribution of the $NPE_{m}$ with sufficient statistics. Figure 7 presents the distribution of $NPE_{m}$ in the case of only one threshold according to all $TOT_{i}^{1}$ values.

By obtaining the mean value of the $NPE_{m}$ ($\langle NPE_{m}\rangle$) and its standard deviation ($\Delta NPE_{m}$) for each set $\tau_{i}$, a conversion table (T) can be constructed (cf. Equation (1)).

(1) $$T:\tau_{i}\to \langle NPE_{m}\rangle \pm \Delta NPE_{m}$$

Within the range of PEs being studied, 10,000 events per PE value are simulated and subsequently converted into $NPE_{m}$ using the table. The relative uncertainty α for each PE value, after conversion, is defined as Equation (2):

(2) $$\alpha =\frac{\sqrt{\langle \Delta NPE_{m}^{2}\rangle }}{\langle NPE_{m}\rangle }$$

**Figure 8:**
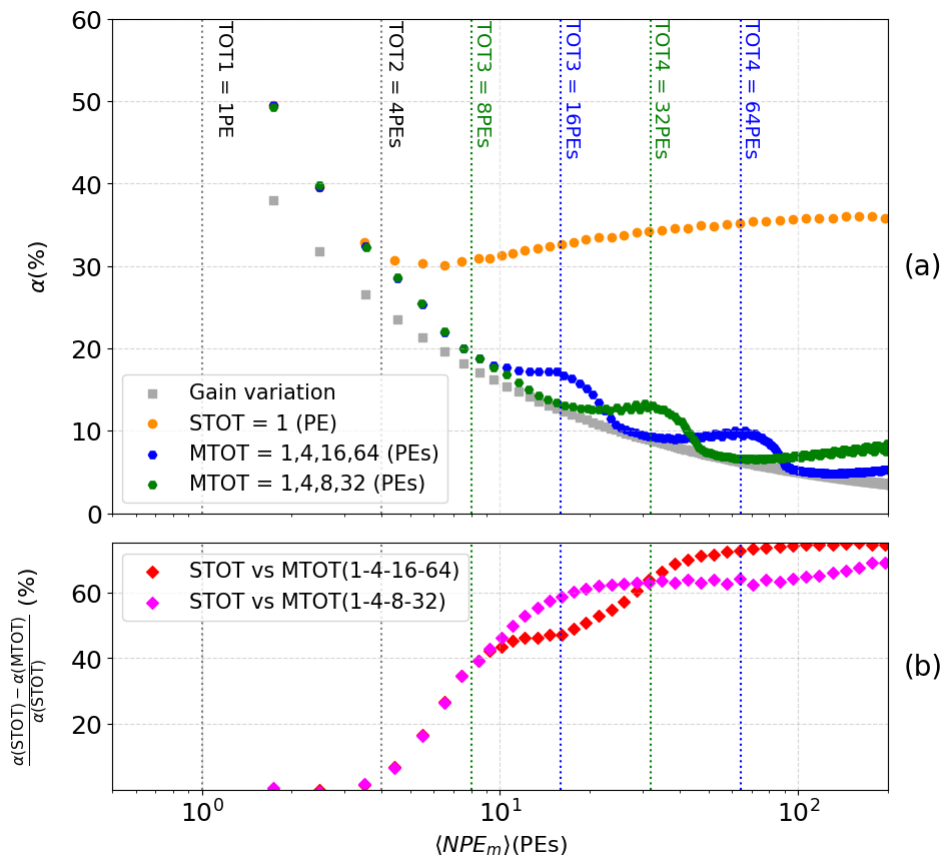
Resolution of the conversion of the charge into the number of PEs as a function of the number of PEs computed with the MTOT method and the STOT in a range from 1 to 200 PEs (**a**). Relative difference between STOT and MTOT (**b**).

It provides an observable to assess the resolution of the MTOT method. Figure 8a shows the MTOT method for two configuration of threshold levels, in blue for (1,4,16,64) PEs an in green for (1,4,8,32) PEs. These resolutions are compared with the one of the STOT method, as well (cf. orange vs blue or green dots in Figure 8a). The theoretical ideal resolution, depicted by gray squares, represents a scenario where the only perturbation is the unavoidable variation in PMT gain, while all other potential disturbances are absent. This comparison highlights the impact of these disturbances on the reconstruction accuracy previously described in Section 4.1. Figure 8b directly compares the two methods: STOT and MTOT. The relative difference between these methods is calculated for both sets of thresholds. This graph significantly highlights the precision improvement provided by MTOT. With each new threshold triggered (represented by blue and green dashed lines), the resolution of the number of $NPE_{m}$ increases dramatically, reaching up to 70% for the highest signals (>64 PEs) with the first set of thresholds within the studied range. It is important to note that the second set (magenta curve) offers better accuracy at the beginning of the interval studied, as its configuration is optimized for detecting smaller signals. The number of PEs detected in XEMIS2 depends on the activity of the radioactive source used. Accurate knowledge of the distribution of VUV photons across the XEMIS2 PMT network as a function of activity will enable maximizing efficiency by selecting the optimal threshold configuration for each injected dose.

### 4.4. Time Correction

**Figure 9:**
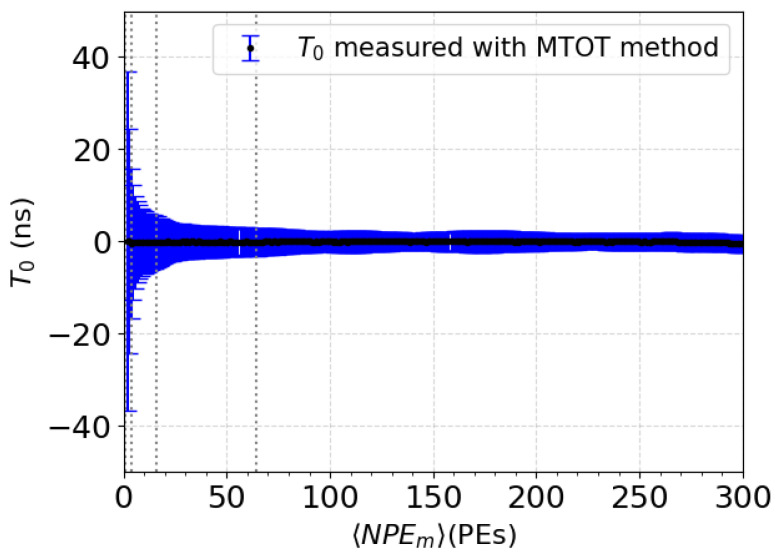
Distribution of $T_{0}$ across a range of 1 to 200 PEs for the set of thresholds 1, 4, 16, and 64 PEs.

The time walk represents the temporal variation in the LE of the signal emitted by the LETD module. This variation depends, in particular, on the amplitude of the PSA signal and, therefore, on the number of PEs. To properly determine the arrival time of the scintillation signal by computing the LE, a time walk correction must be applied. The time walk is computed for each PE value in the considered interval of [1;200]. Assuming that the events are independent for all thresholds, the arrival time $T_{0}$ of the light signal is defined as Equation (3).

(3) $$T_{0}=\frac{\sum_{i=1}^{4}\sigma_{i}^{-2}\left(NPE_{m}\right)\cdot T_{i}^{c}}{\sum_{i=1}^{4}\sigma_{i}^{-2}\left(NPE_{m}\right)}$$

where $T_{0}$ is the time of the reconstructed event, $\sigma_{i}^{2}\left(NPE_{m}\right)$ is the time walk standard variation on threshold *i*, and $T_{i}^{c}$ is the corrected time of the $LE_{i}$. According to the DAQ fluctuation, this $T_{0}$ can variate for a same $NPE_{m}$. This variation is presented in Figure 9. In this figure, each point represents an instance of $T_{0}$ measured using the MTOT method. The standard deviation of the points indicates the extent of variation in the reconstructed time for events with a given $\langle NPE_{m}\rangle$. Notably, the variation is more pronounced at lower $\langle NPE_{m}\rangle$ values, which is consistent with the expected higher time walk error for smaller signals. As $\langle NPE_{m}\rangle$ increases, the variation in $T_{0}$ decreases, demonstrating an improved timing accuracy for higher signals. The vertical dashed lines at specific $\langle NPE_{m}\rangle$ values mark the thresholds of the first set (1-4-16-64 PEs) used in the MTOT method. Figure 9 highlights that the timing precision improves significantly as the number of photoelectrons increases, which is critical for accurate event reconstruction in the XEMIS2 system.

**Figure 10:**
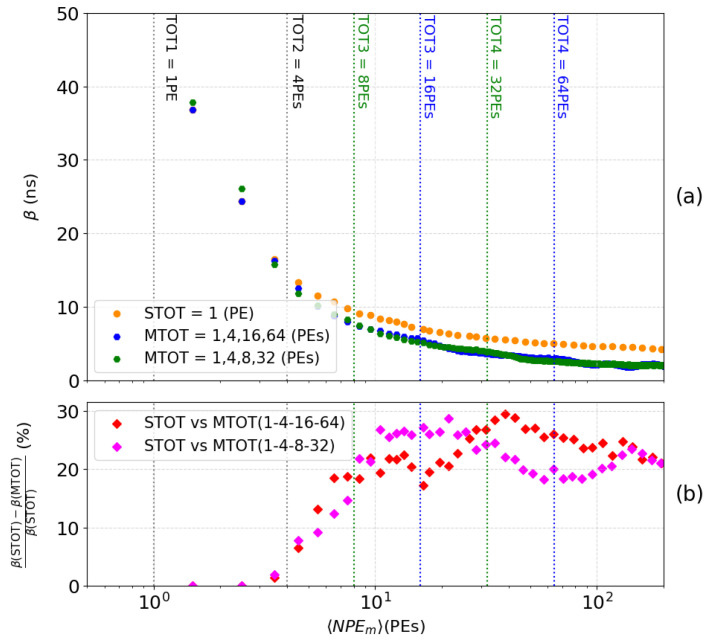
Resolution of the conversion of the event time from the LE computed according to the MTOT and TOT methods within a range of 1 to 200 PEs (**a**). Relative difference between STOT and MTOT (**b**).

Figure 10 shows the standard deviation (represented by β) of the $T_{0}$ distribution. Figure 10a compares the reconstruction of the initial time $T_{0}$ using the STOT methods (yellow curve) and MTOT (blue curve for the set 1, 4, 16, and 64 PEs and green curve for the set 1, 4, 8, and 32 PEs) by plotting the parameter β as a function of $NPE_{m}$. Figure 10b illustrates the relative difference between the two methods for both sets of thresholds, calculated as before. This visualization highlights the improvement in the precision of the reconstruction of the initial time $T_{0}$ provided by the MTOT method. The correction of the walk provided by each threshold allows for an improvement of 20% to 30% in the time reconstruction of the event in the studied range. The interval where the precision is maximized depends on the configuration of the thresholds.

## 5. Conclusions

A new electronic board for reading the scintillation signal based on the MTOT method has been developed for the XEMIS2 camera to improve the interaction time of the γ rays and the number of PEs detected by the PMTs from the scintillation signals collected. The prototype, composed of discrete components, was characterized on a test bench and demonstrated a good performance in measuring the scintillation signal. A simulation was developed to study the behavior of the TOT and LE generated using the MTOT method, taking into account the noise sources present when the XEMIS2 camera operates under real conditions, including the delay between the emission of VUV photons. The simulation shows an overall improvement of up to 70% in the reconstruction of the number of PEs by the front-end electronics of the light acquisition chain using the MTOT method, compared with the STOT method. Additionally, a new signal arrival time correction method has been tested using the four generated LEs, showing a significant increase in the precision of the light signal arrival time reconstruction, ranging from 20% to 30%. These improvements are expected to enable the upgrade of the LXeTPC XEMIS2 camera.

The electronic board is currently in the prototyping phase, composed of discrete elements, and it would be pertinent to consider its integration into an ASIC (Application-Specific Integrated Circuit), as was performed for the Dual TOT [15]. This approach would not only allow for system miniaturization and direct integration of the electronics into the camera, but also improve overall performance by reducing the power consumption, increasing the processing speed, and enhancing the computing accuracy. Furthermore, it would be advisable to develop techniques to combine the MTOT signals to reduce the output data volume, particularly for higher activity levels, as is the case in conventional PET cameras. This optimization would be crucial to ensure the system’s ability to handle larger data flows while maintaining a high measurement accuracy.

## Figures and Tables

**Table 1:** Technical specifications of XSREMTOT electronics.

Properties	Values & Units
Type of trigger	Self-trigger by the LETD module
Number of input channels	2 (PMT + CALIB)
Pulse-shaping amplifier peaking time	50–60 ns
Charge dynamic range	0–16,000 fC
Threshold dynamic range	0–9600 fC
Supply Voltage	±5 V
Maximum current	±1.2 A

## Data Availability

Data are contained within the article.
